# Efficacy of danhong injection adjuvant therapy in patients with acute ischemic stroke: a real-world, multicenter, retrospective study

**DOI:** 10.3389/fphar.2025.1608719

**Published:** 2025-06-12

**Authors:** Danping Pan, Haitong Wan, Yu He, Jiehong Yang, Yilei Guo, Li Yu, Feihu Zhang, Guoqing Zheng, Bin Xu, Yaohong Song, Mingjun Zhao, Xiangzhe Liu, Jianhe Liu, Gang Sun, Yaming Lin

**Affiliations:** ^1^ School of Basic Mecicine Sciences, Zhejiang Chinese Medical University, Hangzhou, China; ^2^ Academy of Chinese Medical Sciences, Henan University of Chinese Medicine, Zhengzhou, China; ^3^ School of Pharmaceutical Sciences, Zhejiang Chinese Medical University, Hangzhou, China; ^4^ Department of Neurology, The First Affiliated Hospital of Zhejiang Chinese Medical University (Zhejiang Provincial Hospital of Chinese Medicine), Hangzhou, China; ^5^ Department of Neurology, The Second Affiliated Hospital of Zhejiang Chinese Medical University, Hangzhou, China; ^6^ Department of Cardiology, Nanjing Hospital of Chinese Medicine Affiliated to Nanjing University of Chinese Medicine, Nanjing, China; ^7^ Department of Cardiology, Affliated Hospital of Shaanxi University of Chinese Medicine, Xianyang, China; ^8^ Department of Encephalology, The First Affiliated Hospital of Henan University of Chinese Medicine, Zhengzhou, China; ^9^ Department of Cardiology, The First Hospital of Hunan University of Chinese Medicine, Changsha, China; ^10^ Department of Cardiology, The First Affiliated Hospital of Guizhou University of Traditional Chinese Medicine, Guiyang, China; ^11^ Department of Neurology, Yunnan Provincial Hospital of Traditional Chinese Medicine, Kunming, China

**Keywords:** danhong injection, acute ischemic stroke, clinical efficacy, real-world multicenter retrospective study, traditional Chinese medicine

## Abstract

**Background:**

Previous clinical and experimental studies indicate that Danhong injection (DHI) confers protective effects against acute ischemic stroke (AIS). However, due to limited sample sizes, large-scale clinical studies are still needed to confirm its efficacy.

**Methods:**

This real-world, multicenter retrospective study used inpatient data from eight centers across Mainland China. AIS patients were divided into a DHI group or a Non-DHI group depending on whether they received DHI (7–14 consecutive days). Propensity score matching (PSM) was applied to balance baseline differences, and multiple analytical methods (crude analysis, multivariate regression, stabilized inverse probability of treatment weighting [sIPTW], and PSM combined with multivariate regression) were conducted. The primary outcome was the NIHSS score at discharge. Secondary outcomes included the proportions of patients with post-treatment NIHSS scores ≤4 or ≤1, the mRS score, the proportion of patients achieving mRS ≤1, and the incidence of in-hospital complications (IHC).

**Results:**

A total of 3,560 patients were enrolled, including 1,425 in the DHI group, and 2,135 in the Non-DHI group, with 1,415 matched pairs identified via PSM. After treatment, the NIHSS score in the DHI group was 2.01 ± 3.10, compared with 2.50 ± 3.26 in the Non-DHI group, indicating significantly lower scores in the DHI group (adjusted RR = 0.81, 95% CI: 0.74–0.88, *P* < 0.001). These findings were consistent across multiple analytical methods (RR = 0.79–0.82). After treatment, the proportion of patients with NIHSS ≤4 and ≤1 was higher in the DHI group (adjusted RR = 1.02, 95% CI: 1.01–1.03, *P* = 0.005; adjusted RR = 1.07, 95% CI: 1.05–1.10, *P* < 0.001). The DHI group also had a lower mRS score (*P* < 0.001) and a higher proportion of patients achieving mRS ≤1 (adjusted RR = 1.12, 95% CI: 1.10–1.15, *P* < 0.001). No noteworthy difference was found between the two groups in the incidence of IHC (adjusted RR = 1.01, 95% CI: 0.99–1.03, *P* = 0.320).

**Conclusion:**

DHI adjunctive therapy may improve neurological outcomes in patients with AIS. However, additional randomized controlled trials (RCTs) are needed to confirm its effectiveness in routine biomedicine-based clinical practice.

**Clinical Trial Registration:**

https://www.chictr.org.cn/bin/project/edit?pid=211769, identifier ChiCTR2400079391.

## Introduction

Globally, stroke is the third most common cause of mortality (10.7% of total deaths) and stands fourth in disability-adjusted life years (DALYs) lost (5.6% of total DALYs) (GBD 2021 Diseases and Injuries Collaborators, 2024; GBD 2021 Stroke Risk Factor Collaborators et al., 2024). In China, stroke imposes a notably high disease burden. According to the latest Global Burden of Disease estimates, 3.94 million new stroke cases were recorded nationwide in 2019, including 2.87 million new Ischemic Stroke (IS) events, and 1.03 million patients died from IS (Wang et al., 2022). Despite guideline-based optimal treatment and secondary prevention strategies, the 1-year stroke recurrence rate remains 9.6%–17.7% (Chinese Society of Neurology, and Chinese Stroke Society, 2022; Pan et al., 2021). In clinical practice across China, traditional Chinese medicine (TCM) is commonly used as an adjunctive therapy for acute ischemic stroke (AIS), largely due to its potential multi-target and multi-pathway effects.

Danhong injection (DHI) is composed of *Salvia miltiorrhiza* (Danshen) and *Carthamus tinctorius* (Honghua), promoting blood circulation, resolving blood stasis, and relaxing meridians. As one of the most commonly used Chinese botanical drug injections in China, DHI is approved for the treatment of ischemic cardiovascular and cerebrovascular diseases (Fu et al., 2018). DHI exhibits multiple pharmacological effects, including inhibiting oxidative stress and inflammation, anticoagulation, lowering lipids, preventing apoptosis, inducing vasodilation, and promoting angiogenesis (Feng et al., 2019). In animal experiments, DHI alleviated neuronal injury in the ischemic penumbra of rats after cerebral ischemia/reperfusion (CI/R) (Zeng et al., 2021). In IS model rats, DHI reduced infarct volume and enhanced neurological recovery by promoting neurogenesis (Li et al., 2023). Moreover, a meta-analysis involving 67 randomized controlled trials (RCTs) indicated that combining DHI with biomedicine significantly enhanced neurological outcomes, self-care capacity, and blood lipid profiles in IS patients (Ma et al., 2022).

Nevertheless, previous clinical research was constrained by relatively small sample sizes and tended to focus on specific endpoints, resulting in insufficient evidence regarding the overall efficacy of DHI as an adjunct therapy in AIS. To our knowledge, no large-scale real-world study has comprehensively evaluated the clinical effectiveness of adjunctive DHI therapy in routine biomedicine-based AIS management. Hence, this real-world, multicenter study set out to evaluate the impact of combining DHI with standard treatment on neurological function and other clinical outcomes in AIS patients, ultimately supplying stronger clinical evidence for DHI’s use as an adjunct therapy.

## Methods

### Study design

This multicenter retrospective cohort study drew on hospital medical records from eight medical centers in Mainland China from June 2018 to May 2023. A list of all participating centers is provided in Supplementary Table S1. The study protocol was granted by the Ethics Committee of the Second Affiliated Hospital of Zhejiang Chinese Medical University (No.2023-072-01) and registered at the Chinese Clinical Trial Registry (ChiCTR2400079391). Reporting adhered to recommended guidelines for observational studies using routinely collected health data (Von Elm et al., 2007; Langan et al., 2018) (Supplementary Table S2). As the dataset uses anonymous identifiers to protect patient privacy, informed consent was not required.

### Study participants

Participants qualified for inclusion if they met the following conditions: (1) Patients discharged with a primary biomedicine-based diagnosis of AIS (Chinese Society of Neurology, and Chinese Stroke Society, 2024), with a disease course of ≤2 weeks; (2) Patients aged ≥40 years; (3) A hospital stay of 7–21 days; (4) Patients who received DHI therapy for 7–14 consecutive days during hospitalization, or did not receive DHI treatment.

Individuals were excluded under any of these conditions: (1) Patients with insufficiently detailed medical records, preventing the extraction of key information; (2) Patients who met the criteria for brain death upon admission or who were discharged against medical advice, died in hospital, or experienced major adverse cardiovascular and cerebrovascular events (MACCE) during hospitalization; (3) Patients who did not receive DHI therapy in accordance with the currently recommended guideline dosage (20 mL once daily, administered intravenously) (Fu et al., 2018).

### Procedures

The exposure factor in this cohort study was whether patients received adjunctive DHI therapy. Those in the DHI group were administered DHI at the guideline-recommended dose (20 mL once daily, given intravenously) for at least seven consecutive days (Fu et al., 2018). Most patients initiated DHI treatment within 48 h of admission. The treatment duration ranged from 7 to 14 days, as determined by the attending physicians. Due to the retrospective nature of the study and variability in clinical practice, no subgroup analysis was conducted based on the timing of initiation or treatment duration.

Both groups received standard biomedicine-based therapy in accordance with existing guidelines (Chinese Society of Neurology, and Chinese Stroke Society, 2024). Baseline characteristics at admission were collected for each patient, including clinical data (Table 1), laboratory tests, and the baseline National Institutes of Health Stroke Scale (NIHSS) score. At discharge, the NIHSS and modified Rankin Scale (mRS) scores were recorded, and the occurrence of in-hospital complications (IHC) was evaluated. The manually extracted data then underwent secondary review and verification to ensure consistency.

**TABLE 1:** Baseline characteristics of patients receiving or not receiving DHI before and after PSM.

Characteristics a	Unmatched patients			Propensity-score-matched patients		
	DHI group (n = 1,425)	Non-DHI group (n = 2,135)	SMD b	DHI group (n = 1,415)	Non-DHI group (n = 1,415)	SMD
Sex, n(%)						
Male	883 (62.0)	1,334 (62.5)	0.011	875 (61.8)	883 (62.4)	0.012
Female	542 (38.0)	801 (37.5)		540 (38.2)	532 (37.6)	
Age, mean (SD)	68.37 (11.84)	67.61 (11.55)	0.065	68.29 (11.82)	68.23 (11.51)	0.005
District, n(%) c						
East	479 (33.6)	594 (27.8)	0.178	469 (33.1)	475 (33.6)	0.011
Middle	476 (33.4)	660 (30.9)		476 (33.6)	477 (33.7)	
West	470 (33.0)	881 (41.3)		470 (33.2)	463 (32.7)	
Smoking status, n(%)						
Current smoker	392 (27.5)	615 (28.8)	0.046	390 (27.6)	392 (27.7)	0.004
Former smoker	110 (7.7)	182 (8.5)		109 (7.7)	108 (7.6)	
Never smoker	923 (64.8)	1,338 (62.7)		916 (64.7)	915 (64.7)	
Drinking, n (%)	345 (24.2)	562 (26.3)	0.049	344 (24.3)	338 (23.9)	0.010
Medical history, n(%) d						
Stroke	525 (36.8)	671 (31.4)	0.114	519 (36.7)	504 (35.6)	0.022
Ischemic stroke	504 (35.4)	620 (29.0)	0.136	498 (35.2)	488 (34.5)	0.015
Heart disease	332 (23.3)	451 (21.1)	0.052	328 (23.2)	337 (23.8)	0.015
Hypertension	1,103 (77.4)	1704 (79.8)	0.059	1,097 (77.5)	1,108 (78.3)	0.019
Type 2 diabetes	508 (35.6)	766 (35.9)	0.005	503 (35.5)	509 (36.0)	0.009
Hyperlipidemia	341 (23.9)	605 (28.3)	0.100	341 (24.1)	343 (24.2)	0.003
Disease course, n(%)						
≤1d	714 (50.1)	1,121 (52.5)	0.048	710 (50.2)	704 (49.8)	0.008
>1d	711 (49.9)	1,014 (47.5)		705 (49.8)	711 (50.2)	
TOAST classification, n(%)						
LAA	357 (25.1)	576 (27.0)	0.068	356 (25.2)	363 (25.7)	0.032
SVO	395 (27.7)	622 (29.1)		393 (27.8)	408 (28.8)	
Other types e	673 (47.2)	937 (43.9)		666 (47.1)	644 (45.5)	
Infarction size, n(%)						
LI	1,079 (75.7)	1,562 (73.2)	0.059	1,071 (75.7)	1,072 (75.8)	0.002
FLI	346 (24.3)	573 (26.8)		344 (24.3)	343 (24.2)	
Baseline NIHSS score, n(%)						
≤4	1,008 (70.7)	1,528 (71.6)	0.019	1,005 (71.0)	1,006 (71.1)	0.002
5–20	402 (28.2)	586 (27.4)		395 (27.9)	394 (27.8)	
≥21	15 (1.1)	21 (1.0)		15 (1.1)	15 (1.1)	

^a^ Values are presented as n (%) or mean (SD).

^b^ The SMD, was used to compare characteristics between the DHI, and Non-DHI, groups, with an SMD <0.1 indicating balanced and comparable covariates.

^c^ “District” refers to Eastern (Zhejiang, Nanjing), Central (Shaanxi, Henan, Hunan), or Western (Guizhou, Yunnan) regions.

^d^ “Stroke” includes ischemic stroke and hemorrhagic stroke; “Heart disease” includes coronary artery disease, myocardial infarction, atrial fibrillation, and heart failure.

^e^ “Other types” refers to all AIS, patients other than LAA and SVO.

Abbreviations: DHI, danhong injection; SMD, standardized mean difference; SD, standard deviation; LAA, large-artery atherosclerosis; SVO, small-vessel occlusion; LI, lacunar infarction; FLI, focal or large-area infarction; NIHSS, national institutes of health stroke scale.

### Description of DHI

DHI is a standardized Chinese herbal formulation composed of *Salvia miltiorrhiza* Bunge [Lamiaceae; *Salviae miltiorrhizae radix et rhizoma*] and *Carthamus tinctorius* L. [Asteraceae; *Carthami flos*], both of which are listed in the Chinese Pharmacopoeia (2020 edition). The preparation used in this study was a finished commercial product approved by the China Food and Drug Administration (CFDA, Approval No. Z20026866). The HPLC profile of DHI has been reported previously (Li et al., 2023) and provides quantitative information on its representative metabolites.

### Outcomes

The primary outcome was the NIHSS score at discharge (approximately 14 ± 7 days post-treatment). Secondary outcomes included the proportions of patients with NIHSS scores ≤4 and ≤1 at discharge, the mRS score, the proportion of patients with an mRS score ≤1, and the incidence of IHC. The NIHSS measures the severity of neurological deficits in AIS, with ≤4 indicating mild stroke, 4–21 indicating moderate stroke, and ≥21 indicating severe stroke. The mRS evaluates neurological recovery following AIS, ranging from 0 (no residual stroke symptoms) to 6 (death). IHC encompass pneumonia, urinary tract infection, cerebral edema or intracranial hypertension, and deep vein thrombosis or embolism.

### Covariates

To examine the association between DHI adjunct therapy and outcomes, we collected multiple covariates: baseline sex, age, smoking status, drinking, past medical history (IS, stroke, heart disease, hypertension, diabetes, hyperlipidemia), disease course, TOAST classification (large-artery atherosclerosis [LAA], small-vessel occlusion [SVO], other types), laboratory parameters, infarction size (lacunar infarction [LI], focal or large-area infarction [FLI]), and the baseline NIHSS score. Given potential differences in healthcare levels across regions, the study center was included as a confounding factor. Detailed information on all 27 covariates is provided in Supplementary Table S3.

### Statistical analyses

Propensity score matching (PSM) was employed to balance baseline variables between the DHI and Non-DHI groups. A multivariable logistic regression model was used to estimate propensity scores, incorporating previously identified covariates (Kainz et al., 2017). Patients were matched on a 1:1 basis via nearest neighbor matching with a caliper width equal to 0.2 times the standardized difference (SD) of the logit-transformed propensity score (caliper = 0.2 × SD [logit (PS)]) (Makhnevich et al., 2024). A fast matching algorithm was applied without replacement. For both pre- and post-matching datasets, continuous variables were reported as mean ± standard deviation, and categorical variables were presented as frequency (percentage). The standardized mean difference (SMD) was used to compare intergroup differences in baseline characteristics before and after matching, with an SMD <0.1 indicating adequate balance and comparability.

After PSM, outcomes were analyzed according to their data type. For discrete outcomes (e.g., NIHSS score), Poisson regression was first used to calculate the risk ratio (RR) and 95% confidence interval (CI). If overdispersion was detected, negative binomial regression was applied instead (Schober and Vetter, 2021). For binary outcomes (e.g., NIHSS score ≤4), the RR and 95% CI were derived using modified Poisson regression (Zou, 2004). A two-sided P-value <0.05 was deemed statistically significant. As this study was exploratory, no adjustments were made for multiple comparisons. Aside from laboratory parameters, which were <20% missing and recategorized as dichotomous variables, all variables in this study were complete. Detailed information regarding missing data is presented in Supplementary Table S4, and multiple imputation was performed to address these missing values.

To further clarify the relationship between non-random DHI therapy and NIHSS scores, we applied several analytical methods: crude analysis, multivariable regression, and propensity score–based approaches (PSM, stabilized inverse probability of treatment weighting [sIPTW], and PSM combined with multivariable regression) (Geleris et al., 2020). Except for the crude analysis, all other methods incorporated the covariates from Model two for adjustment. We also generated a forest plot to visualize the incidence of post-treatment “NIHSS score >4” across key clinical subgroups, including demographic factors such as age and sex. Other sensitivity analyses included: (1) Fitting multiple PSM models. Model one included sex, age, district, lifestyle factors, and past medical history. Model two added disease course, TOAST classification, infarction size, and baseline NIHSS score. Model three further incorporated laboratory parameters. Model two served as the primary analysis model. (2) Excluding patients who underwent intravenous thrombolysis or endovascular intervention. (3) Excluding patients with severe stroke. (4) Performing a *post hoc* E-value analysis to gauge how large an unmeasured confounder must be to eliminate the observed association (Haneuse et al., 2019). All statistical analyses were performed using R software (version 4.2.1).

## Results

### Patients and baseline characteristics

**FIGURE 1:**
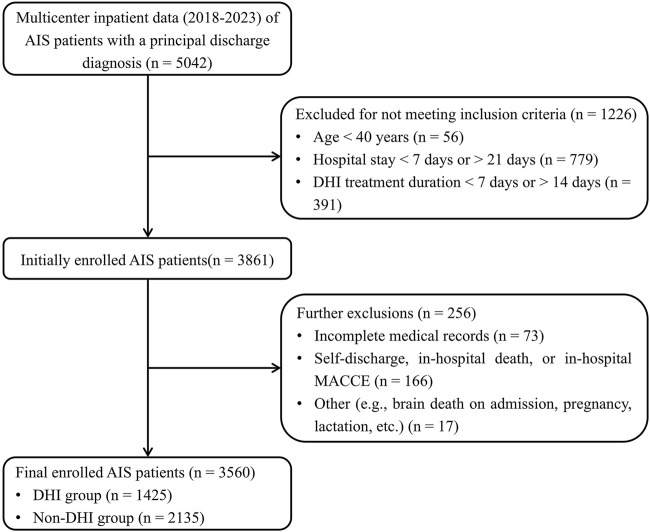
Flow diagram of the study selection process. MACCE includes ischemic stroke, hemorrhagic stroke, myocardial infarction, and vascular-related mortality. Abbreviations: AIS, acute ischemic stroke; DHI, Danhong Injection; MACCE, major adverse cardiovascular and cerebrovascular events.

A total of 5,042 patients discharged with a primary diagnosis of AIS from eight medical centers in Mainland China between June 2018 and May 2023 were screened. Of these, 3,816 met the inclusion criteria. After applying the exclusion criteria, 3,560 patients were ultimately included in the analysis, comprising 1,425 in the DHI group and 2,135 in the Non-DHI group (Figure 1).

**FIGURE 2:**
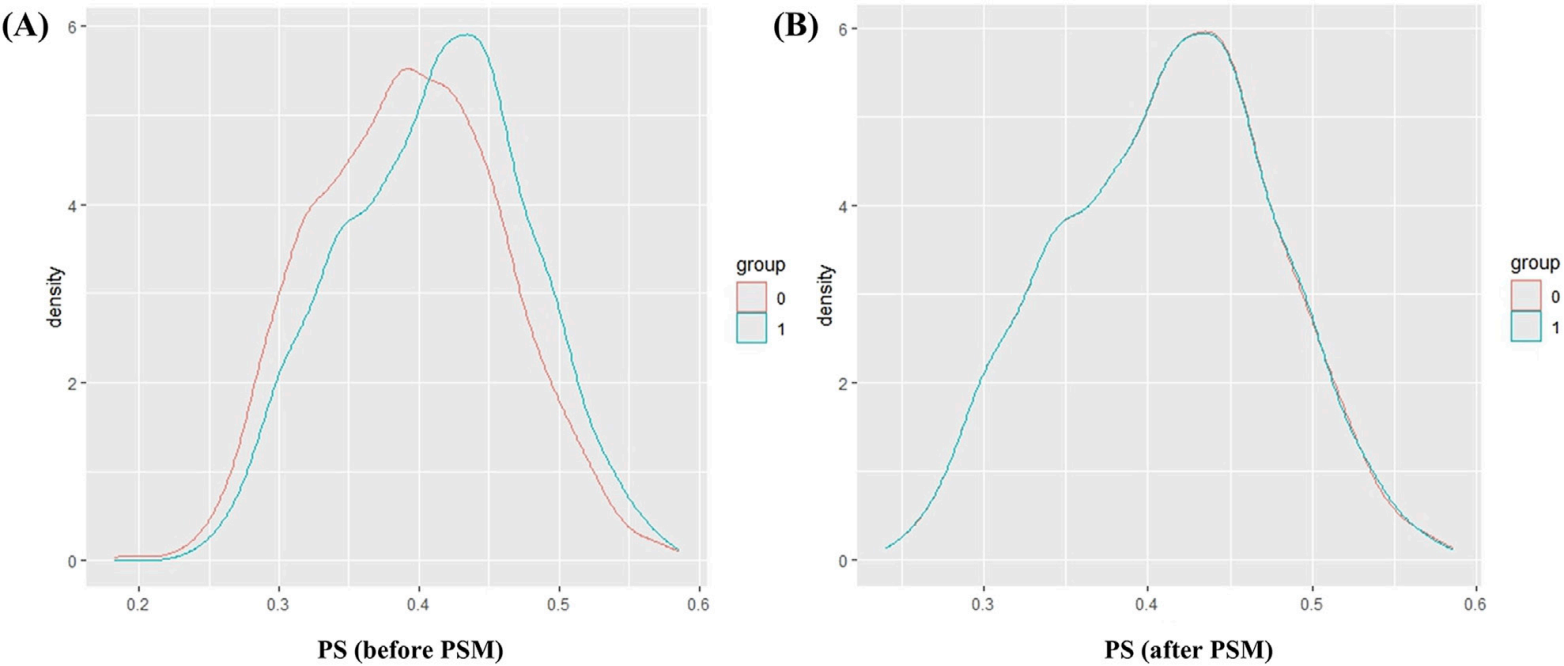
Density plots of propensity scores before and after PSM. **(A)** PS density curve before PSM; **(B)** PS density curve after PSM. Abbreviations: PS, propensity score; PSM, propensity score matching.

The baseline characteristics of patients in the DHI and Non-DHI groups are presented in Table 1 (a full listing of the 27 characteristics is available in Supplementary Table S3). Before matching, all SMD values were below 0.1 except for district, stroke, ischemic stroke, and hyperlipidemia. After matching, all baseline characteristics (1,415 patients per group) were balanced between the DHI and Non-DHI cohorts (SMD <0.1). As a result of PSM, the predicted likelihood distribution (represented by propensity scores) was similar in both groups (Figure 2).

### Primary outcome

In the primary analysis conducted after PSM, the NIHSS score in the DHI group was 2.01 ± 3.10, compared with 2.50 ± 3.26 in the Non-DHI group. Negative binomial regression revealed that the NIHSS score in the DHI group was significantly lower (adjusted RR = 0.81, 95% CI: 0.74–0.88, *P* < 0.001), corresponding to a mean difference (MD) of 0.61 ± 2.81 between the two groups (Table 2).

**TABLE 2:** Association between DHI use and the primary outcomes.

Analysis	DHI group a	Non-DHI group a	RR (95% CI)	p-value	MD b
PSM	2.01 (3.10)	2.50 (3.26)	0.81 (0.74–0.88)	<0.001	0.61 (2.81)
Crude analysis	2.02 (3.10)	2.45 (3.13)	0.82 (0.76–0.89)	<0.001	0.62 (2.66)

^a^ Shown is the post-treatment NIHSS, score, presented as mean (SD).

^b^ MD is calculated as the difference in NIHSS (pre-to post-treatment) for the DHI, group minus the difference in NIHSS (pre-to post-treatment) for the Non-DHI, group, and is also presented as mean (SD).

Abbreviations: DHI, danhong injection; RR, risk ratio; CI, confidence interval; MD, mean difference; PSM, propensity score matching.

In the unadjusted (crude) analysis, the post-treatment NIHSS score was 2.02 ± 3.10 in the DHI group and 2.45 ± 3.13 in the Non-DHI group. Univariate negative binomial regression again showed the DHI group’s NIHSS score was significantly lower (adjusted RR = 0.82, 95% CI: 0.76–0.89, *P* < 0.001), with an MD of 0.62 ± 2.66 (Table 2). Further multivariable and propensity score analyses showed consistent findings (RR = 0.79–0.82) (Table 3).

**TABLE 3:** Associations between DHI use and the primary outcome in crude, multivariable, and propensity-score analyses.

Analysis	RR (95% CI)
Crude analysis	0.82 (0.76–0.89)
Multivariable analysis a	0.79 (0.75–0.84)
Propensity-score analyses	
With matching b	0.81 (0.74–0.88)
With sIPTW c	0.81 (0.74–0.90)
With matching and multivariable analysis d	0.79 (0.74–0.83)

^a^ The RR, is derived from a multivariable negative binomial regression model adjusted for 15 covariates (Table 1). Analysis includes all 3,560 patients.

^b^ This primary analysis uses a univariable negative binomial regression model where 15 covariates are matched according to the propensity score. It includes 2,830 patients (1,415 receiving DHI, 1,415 not).

^c^ The RR, is obtained from a univariable negative binomial regression model applying sIPTW, to the same 15 covariates, covering all patients.

^d^ The RR, is generated from a multivariable negative binomial regression model using the same 15 covariates matched by the propensity score, analyzing 2,830 patients.

Abbreviations: RR, risk ratio; CI, confidence interval; sIPTW, stable inverse probability of treatment weighting.

### Secondary outcome

**FIGURE 3:**
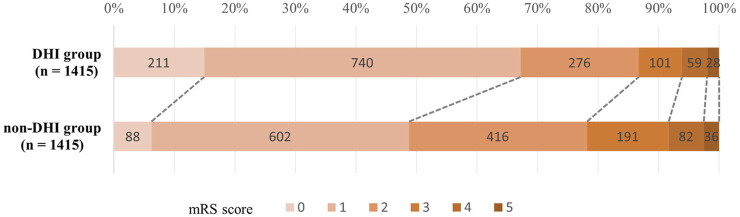
Distribution of mRS scores in AIS patients.

Among the 2,830 patients after PSM (1,415 in the DHI group and 1,415 in the Non-DHI group), the proportion of patients with NIHSS scores ≤4 and ≤1 were significantly higher in the DHI group (adjusted RR = 1.02, 95% CI: 1.01–1.03, *P* = 0.005; adjusted RR = 1.07, 95% CI: 1.05–1.10, *P* < 0.001). The DHI group also had significantly lower mRS score than the Non-DHI group (*P* < 0.001) (Figure 3), and a greater proportion of patients achieved mRS ≤1 (adjusted RR = 1.12, 95% CI: 1.10–1.15, *P* < 0.001). No significant differences were observed in the incidence of IHC or pneumonia between the two groups (adjusted RR = 1.01, 95% CI: 0.99–1.03, *P* = 0.320; adjusted RR = 1.01, 95% CI: 0.99–1.02, *P* = 0.478) (Table 4).

**TABLE 4:** Association between DHI use and the secondary outcomes.

Secondary outcomes	DHI group (n = 1,415)	Non-DHI group (n = 1,415)	Adjusted RR (95% CI)	p-value
NIHSS score ≤4, n (%)	1,271 (89.82)	1,223 (86.43)	1.02 (1.01–1.03)	0.005
NIHSS score ≤1, n (%)	813 (57.46)	659 (46.57)	1.07 (1.05–1.10)	<0.001
mRS score ≤1, n (%)	951 (67.21)	690 (48.76)	1.12 (1.10–1.15)	<0.001
IHC, n (%)	174 (12.30)	157 (11.10)	1.01 (0.99–1.03)	0.320
Pneumonia, n (%)	138 (9.75)	127 (8.98)	1.01 (0.99–1.02)	0.478

PSM, was used to balance baseline differences between the DHI, and Non-DHI, groups. The *p*-value indicates the comparison between these two groups. Abbreviations: DHI, danhong injection; RR, risk ratio; CI, confidence interval; IHC, in-hospital complications.

#### Subgroup and sensitivity analyses

**FIGURE 4:**
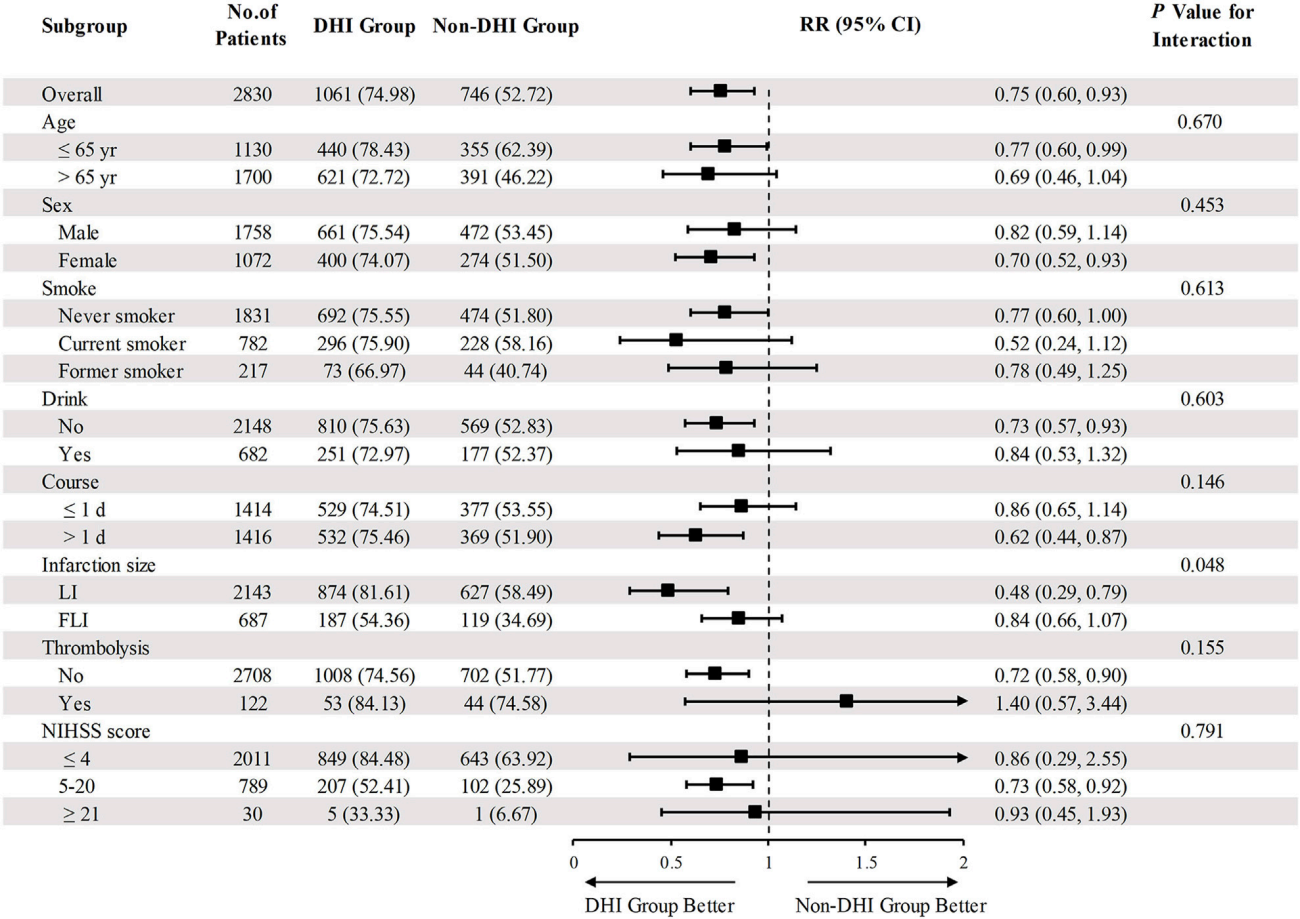
Subgroup analysis of the relationship between DHI use and “NIHSS score >4” in AIS patients. Abbreviations: DHI, Danhong Injection; RR, risk ratio; CI, confidence interval; LI, lacunar infarction; FLI, focal or large-area infarction.

Patients with NIHSS score >4 are generally considered to have moderate-to-severe stroke, indicating substantial neurological impairment that may affect prognosis. A *post hoc* subgroup analysis evaluated the incidence of NIHSS score >4 in different AIS subgroups after treatment. The findings revealed that the DHI group had an overall lower relative risk, demonstrating consistent effectiveness across multiple subgroups (Figure 4). Apart from infarction size (*P* = 0.048), the interaction *P*-values for all other subgroups were nonsignificant (*P* > 0.05), indicating that infarct size significantly modulates the risk of having an NIHSS score >4. Notably, compared with the FLI subgroup, the LI subgroup had a markedly reduced relative risk.

Multiple *post hoc* sensitivity analyses—including fitting various PSM models, excluding patients who underwent intravenous thrombolysis or endovascular intervention, and excluding those with severe stroke—produced similar results (Supplementary Table S5). The E-value for the RR was 1.77, and the upper confidence bound for the primary outcome was 1.53, indicating that a strong unmeasured confounder would be required to invalidate the observed association or its 95% CI (Supplementary Figure S1).

## Discussion

To our knowledge, this is the first real-world, multicenter, retrospective cohort study to evaluate the efficacy of adjunctive DHI therapy in patients with AIS. Of the 3,560 patients with complete data on both primary and secondary outcomes, 2,830 (1,415 in each group) were successfully matched by PSM. These findings suggest that adjunctive DHI therapy not only lowers post-treatment NIHSS scores and increases the proportion of patients achieving NIHSS ≤4 or ≤1, but also reduces mRS scores and raises the proportion of patients with mRS ≤1. Hence, it appears to enhance both neurological function and quality of life. Notably, no effect of DHI on IHC was observed in AIS patients.

DHI is prepared from *Salvia miltiorrhiza* (Danshen) and *Carthamus tinctorius* (Honghua), and is commonly used in Chinese clinical practice to treat ischemic cardiovascular and cerebrovascular diseases due to its blood-activating and stasis-resolving properties. According to the literature, DHI contains multiple active metabolites, including danshensu, salvianolic acids, and hydroxysafflor yellow A (HSYA), which act on diverse molecular targets to improve hemorheology, protect vascular endothelium, and attenuate inflammation and oxidative stress (Feng et al., 2019; Ma et al., 2022). Experimental studies have shown that DHI reduces infarct volume, promotes neurovascular remodeling, and facilitates neurological recovery in IS models (Feng et al., 2019). These effects are thought to be mediated by suppression of the NF-κB and NLRP3 inflammasome pathways (Du et al., 2021; Wang J. et al., 2024), enhancement of antioxidant capacity via the Nrf2/ARE signaling axis (Guo et al., 2014), and regulation of neuronal apoptosis and autophagy through the PI3K/Akt/mTOR and BDNF/CREB pathways (Zhang et al., 2023; Li et al., 2023). Among its bioactive metabolites, danshensu exhibits anti-inflammatory and autophagy-regulating effects (Hu et al., 2022); salvianolic acids inhibit oxidative stress and platelet aggregation (Zhao et al., 2024; Liu et al., 2023); and HSYA exerts neuroprotective actions, potentially by modulating SIRT1-related pathways (Fangma et al., 2021). Collectively, these multi-target mechanisms support the therapeutic potential of DHI in the treatment of AIS.

Building on these advantages, these studies have explored the efficacy of DHI in patients with AIS and angina. They found that DHI improved neurological function and self-care ability in AIS patients in a dose-dependent manner (Du et al., 2018; Ma et al., 2022). In angina patients, add-on DHI therapy reduced the frequency of anginal episodes, alleviated myocardial ischemic symptoms, and improved TCM syndromes (Liu et al., 2021; Chen et al., 2022). These reports further indicated that adjunct DHI therapy did not introduce additional safety risks. However, their conclusions relied mainly on relatively small sample sizes, limiting the ability to fully characterize DHI’s effectiveness in real-world practice.

The results of our study align with outcomes reported in other adjunctive therapy trials. For example, the TISS trial indicated that adding Tongxinluo markedly increased the proportion of AIS patients attaining a 90-day NIHSS score of 0–1 or a reduction of ≥4 points (Dong et al., 2024). In the TASTE trial, Edaravone Dexborneol lowered the NIHSS score by an additional 0.4 points compared with Edaravone alone in the acute phase (Xu et al., 2021). The BAST trial showed that at 90 days, butylphthalide reduced the NIHSS score by about one point more than the control group (Wang et al., 2023). Using a large-scale, multicenter, real-world cohort, our study similarly confirmed the beneficial effects of DHI on NIHSS and mRS scores, supporting its role in adjunctive AIS treatment. However, whether these short-term improvements in neurological function and disability reduction translate into better long-term outcomes remains unclear and will require extended follow-up and large-scale RCTs.

This study used a multicenter, retrospective, real-world cohort design, providing data closer to clinical practice, including treatment pathways, concomitant medications, and disease heterogeneity, than conventional RCTs. It thereby offers greater external validity for applying botanical drug–based therapy in AIS (Tan et al., 2022). In contrast to prior RCTs or preclinical reports, this multicenter analysis provides pragmatic and clinically relevant evidence on DHI use in real-world AIS patients across diverse hospital settings. Nonetheless, real-world observational studies may still be influenced by unaccounted confounding factors (Psaty et al., 1999). To mitigate potential confounding, we conducted crude analysis, PSM, sIPTW, and multivariable regression. Subgroup and sensitivity analyses were then performed to verify the robustness and consistency of our findings. No effect of adjunct DHI therapy on IHC was identified, possibly because patients who develop such complications often present with advanced age, severe stroke, or multi-organ dysfunction.

As this was a retrospective study, active monitoring of medication-related adverse events (AEs) was not performed. Nonetheless, large-scale pharmacovigilance studies in China have reported a low incidence of DHI-related adverse drug reactions (ADRs), approximately 3.5‰, primarily type A reactions (e.g., sweating, dizziness, headache, flushing), which typically resolved after drug withdrawal (Liu et al., 2021; Chen et al., 2022). Notably, no cases of anaphylactic shock or allergic respiratory distress were reported. Moreover, RCTs have reported no significant difference in AEs incidence between DHI-treated and control groups (Li et al., 2015; Ge et al., 2022). DHI has also shown good compatibility and safety when administered with common intravenous solvents (Wang A. et al., 2024). These findings collectively support the favorable safety profile of DHI in both clinical trials and routine practice.

This large-scale, multicenter study closely reflects real-world TCM clinical settings, offering a model for examining adjunctive AIS therapy. Although high-quality evidence supporting TCM interventions in AIS remains limited, TCM’s multi-component and multi-target properties suggest broader clinical utility. By evaluating DHI’s impact on neurological function and disability in AIS patients, our results confirm that adjunct DHI therapy can improve clinical outcomes.

Nonetheless, our study has certain limitations. First, although we used multiple approaches to address confounding, unmeasured factors (e.g., prior medication use) may still bias the results, and E-value analysis only provides supplementary insights. Second, excluding AIS patients hospitalized for longer than 21 days reduced the representation of severely ill patients requiring extended hospitalization. Third, our findings were limited to short-term clinical outcomes (discharge NIHSS and mRS scores), as follow-up imaging, biomarker, and other relevant data were not consistently available across centers, making it infeasible to assess radiographic or mechanistic responses. Fourth, all eight participating centers were affiliated with TCM institutions, potentially limiting generalizability. Fifth, as a retrospective observational study without randomization or blinding, there may be a risk of residual bias, though we applied multiple statistical strategies to enhance internal validity and transparency. In light of these limitations, our results should be interpreted with caution. Looking ahead, well-designed multicenter RCTs are needed to validate the efficacy of adjunctive DHI therapy and to provide stronger evidence for its clinical application in AIS.

## Conclusion

In conclusion, adjunctive DHI therapy can lower NIHSS and mRS scores in AIS patients, thereby enhancing clinical outcomes. These findings support the use of DHI as an adjunctive treatment for AIS. However, larger-scale, multicenter RCTs are still needed to further validate its efficacy and safety in routine biomedicine-based practice.

## Data Availability

The original contributions presented in the study are included in the article/Supplementary Material, further inquiries can be directed to the corresponding author.

## Abbreviations

DALYs, disability-adjusted life years; IS, Ischemic Stroke; TCM, traditional Chinese medicine; AIS, acute ischemic stroke; DHI, Danhong injection; CI/R, cerebral ischemia/reperfusion; RCTs, randomized controlled trials; MACCE, major adverse cardiovascular and cerebrovascular events; NIHSS, National Institutes of Health Stroke Scale; mRS, modified Rankin Scale; IHC, in-hospital complications; LAA, large-artery atherosclerosis; SVO, small-vessel occlusion; LI, lacunar infarction; FLI, focal or large-area infarction; PSM, Propensity score matching; SD, standardized difference; SMD, standardized mean difference; RR, risk ratio; CI, confidence interval; sIPTW, stabilized inverse probability of treatment weighting; MD, mean difference; HSYA, hydroxysafflor yellow A; AEs, adverse events.
