# Supplementary material for: Efficacy of danhong injection adjuvant therapy in patients with acute ischemic stroke: a real-world, multicenter, retrospective study
Source: Front Pharmacol. 2025 Jun 12;16:1608719. doi: 10.3389/fphar.2025.1608719 (PMC12198127; doi:10.3389/fphar.2025.1608719)
Supplement: Supplementary file 1 [file DataSheet1.docx]

**Supplementary Materials:**

**Table S1** Medical centers participating in the study.

**Table S2** Checklist of recommendations for reporting observational studies using the RECORD Guideline.

**Table S3** All 27 baseline characteristics of patients receiving or not receiving DHI before and after propensity-score matching (Model 3).

**Table S4** Detailed definitions and amount of missing data.

**Table S5** Sensitivity analysis of the associations between DHI use and the primary outcome.

**Table S6** Baseline characteristics of patients receiving or not receiving DHI after sIPTW.

**Table S7** Baseline characteristics of patients receiving or not receiving DHI before and after propensity-score matching (Model 1).

**Table S8** Baseline characteristics before and after PSM, excluding patients who received intravenous thrombolysis or endovascular therapy (Model 4).

**Table S9** Baseline characteristics before and after PSM, excluding patients with severe stroke (baseline NIHSS score ≥ 21) (Model 5).

**Figure S1** Post-hoc E-value analysis to assess the extent of unmeasured confounding that would be required to negate the observed results.

**Figure S2** Ethics approval document.

**Table S1** Medical centers participating in the study.

| No. | Centers |
| --- | --- |
| 1 | The First Affiliated Hospital of Zhejiang Chinese Medical University |
| 2 | The Second Affiliated Hospital of Zhejiang Chinese Medical University |
| 3 | Nanjing Hospital of Chinese Medicine Affiliated to Nanjing University of Chinese Medicine |
| 4 | Affliated Hospital of Shaanxi University of Chinese Medicine |
| 5 | The First Affiliated Hospital of Henan University of Chinese Medicine |
| 6 | The First Hospital of Hunan University of Chinese Medicine |
| 7 | The First Affiliated Hospital of Guizhou University of Traditional Chinese Medicine |
| 8 | Yunnan Provincial Hospital of Traditional Chinese Medicine |

**Table S2** Checklist of recommendations for reporting observational studies using the RECORD Guideline.

|  | **Item No** | **Recommendation** | **Reported** |
| --- | --- | --- | --- |
| **Title and abstract** | 1 | (a) Indicate the study’s design with a commonly used term in the title or the abstract | Title;  Abstract |
|  |  | (b) Provide in the abstract an informative and balanced summary of what was done and what was found | Abstract |
| **Introduction** | | | |
| Background/rationale | 2 | Explain the scientific background and rationale for the investigation being reported | Introduction |
| Objectives | 3 | State specific objectives, including any prespecified hypotheses | Introduction |
| **Methods** | | | |
| Study design | 4 | Present key elements of study design early in the paper | Methods - study design |
| Setting | 5 | Describe the setting, locations, and relevant dates, including periods of recruitment, exposure, follow-up, and data collection | Methods - study design, study participants, procedures |
| Participants | 6 | (a) Give the eligibility criteria, and the sources and methods of selection of participants. | Methods - study design, study participants, procedures |
|  |  | (b) For matched studies, give matching criteria and number of exposed and unexposed | Methods - statistical analyses; Results - patients and baseline characteristics |
| Variables | 7 | Clearly define all outcomes, exposures, predictors, potential confounders, and effect modifiers. Give diagnostic criteria, if applicable | Methods - outcomes, covariates |
| Data sources/ measurement | 8 | For each variable of interest, give sources of data and details of methods of assessment (measurement). Describe comparability of assessment methods if there is more than one group | Methods - study design |
| Bias | 9 | Describe any efforts to address potential sources of bias | Methods - statistical analyses;  Discussion |
| Study size | 10 | Explain how the study size was arrived at | Not applicable |
| Quantitative variables | 11 | Explain how quantitative variables were handled in the analyses. If applicable, describe which groupings were chosen and why | Methods - statistical analyses |
| Statistical methods | 12 | (a) Describe all statistical methods, including those used to control for confounding | Methods - statistical analyses |
|  |  | (b) Describe any methods used to examine subgroups and interactions | Methods - statistical analyses |
|  |  | (c) Explain how missing data were addressed | Methods - statistical analyses |
|  |  | (d) If applicable, explain how loss to follow-up was addressed | Not applicable |
|  |  | (e) Describe any sensitivity analyses | Methods - statistical analyses |
| **Results** | | | |
| Participants | 13 | (a) Report numbers of individuals at each stage of study—eg numbers potentially eligible, examined for eligibility, confirmed eligible, included in the study, completing follow-up, and analyzed | Result - patients and baseline characteristics; Figure 1 |
|  |  | (b) Give reasons for non-participation at each stage | Figure 1 |
|  |  | (c) Consider use of a flow diagram | Figure 1 |
| Descriptive data | 14 | (a) Give characteristics of study participants (e.g. demographic, clinical, social) and information on exposures and potential confounders | Result - patients and baseline characteristics; Table 1, Table S2 |
|  |  | (b) Indicate number of participants with missing data for each variable of interest | Methods - statistical analyses; Table S3 |
|  |  | (c) Summarize follow-up time (e.g. average and total amount) | Not applicable |
| Outcome data | 15 | Report numbers of outcome events or summary measures | Results - primary outcome, secondary outcome; Table 2, Table 4 |
| Main results | 16 | (a) Give unadjusted estimates and, if applicable, confounder-adjusted estimates and their precision (e.g. 95% confidence interval). Make clear which confounders were adjusted for and why they were included | Results - primary outcome; Table 2, Table 3 |
|  |  | (b) Report category boundaries when continuous variables were categorized | Result - patients and baseline characteristics; Table 1, Table S2 |
|  |  | (c) If relevant, consider translating estimates of relative risk into absolute risk for a meaningful time period | Not applicable |
| Other analyses | 17 | Report other analyses done—e.g. analyses of subgroups and interactions, and sensitivity analyses | Result - subgroup and sensitivity analyses; Figure 4, Table S4, Figure S3 |
| **Discussion** | | | |
| Key results | 18 | Summarize key results with reference to study objectives | Discussion |
| Limitations | 19 | Discuss limitations of the study, taking into account sources of potential bias or imprecision. Discuss both direction and magnitude of any potential bias | Discussion |
| Interpretation | 20 | Give a cautious overall interpretation of results considering objectives, limitations, multiplicity of analyses, results from similar studies, and other relevant evidence | Discussion |
| Generalizability | 21 | Discuss the generalizability (external validity) of the study results | Discussion |
| **Other information** | | | |
| Funding | 22 | Give the source of funding and the role of the funders for the present study and, if applicable, for the original study on which the present article is based | Funding |

**Table S3** All 27 baseline characteristics of patients receiving or not receiving DHI before and after propensity-score matching (Model 3).

| **Characteristics** a | Unmatched patients | | | Propensity-score-matched patients | | |
| --- | --- | --- | --- | --- | --- | --- |
|  | DHI group  (n = 1425) | Non-DHI group  (n = 2135) | SMD b | DHI group  (n = 1397) | Non-DHI group  (n = 1397) | SMD |
| **Sex, n(%)** |  | | | | | |
| Male | 883 (62.0) | 1334 (62.5) | 0.011 | 867 (62.1) | 855 (61.2) | 0.018 |
| Female | 542 (38.0) | 801 (37.5) |  | 530 (37.9) | 542 (38.8) |  |
| **Age, mean (SD)** | 68.37 (11.84) | 67.61 (11.55) | 0.065 | 68.17 (11.79) | 68.23 (11.34) | 0.006 |
| **District, n(%)** c |  | | | | | |
| East | 479 (33.6) | 594 (27.8) | 0.178 | 455 (32.6) | 465 (33.3) | 0.015 |
| Middle | 476 (33.4) | 660 (30.9) |  | 472 (33.8) | 467 (33.4) |  |
| West | 470 (33.0) | 881 (41.3) |  | 470 (33.6) | 465 (33.3) |  |
| **Smoking status, n(%)** |  | | | | | |
| Current smoker | 392 (27.5) | 615 (28.8) | 0.046 | 382 (27.3) | 383 (27.4) | 0.030 |
| Former smoker | 110 (7.7) | 182 (8.5) |  | 108 (7.7) | 97 (6.9) |  |
| Never smoker | 923 (64.8) | 1338 (62.7) |  | 907 (64.9) | 917 (65.6) |  |
| **Drinking, n (%)** | 345 (24.2) | 562 (26.3) | 0.049 | 340 (24.3) | 332 (23.8) | 0.013 |
| **Medical history, n(%)** d |  | | | | | |
| Stroke | 525 (36.8) | 671 (31.4) | 0.114 | 506 (36.2) | 500 (35.8) | 0.009 |
| Ischemic stroke | 504 (35.4) | 620 (29.0) | 0.136 | 485 (34.7) | 481 (34.4) | 0.006 |
| Heart disease | 332 (23.3) | 451 (21.1) | 0.052 | 322 (23.0) | 315 (22.5) | 0.012 |
| Hypertension | 1103 (77.4) | 1704 (79.8) | 0.059 | 1081 (77.4) | 1084 (77.6) | 0.005 |
| Type 2 diabetes | 508 (35.6) | 766 (35.9) | 0.005 | 496 (35.5) | 487 (34.9) | 0.013 |
| Hyperlipidemia | 341 (23.9) | 605 (28.3) | 0.100 | 337 (24.1) | 340 (24.3) | 0.005 |
| **Disease course, n(%)** |  | | | | | |
| ≤ 1d | 714 (50.1) | 1121 (52.5) | 0.048 | 698 (50.0) | 694 (49.7) | 0.006 |
| > 1d | 711 (49.9) | 1014 (47.5) |  | 699 (50.0) | 703 (50.3) |  |
| **TOAST classification, n(%)** |  | | | | | |
| LAA | 357 (25.1) | 576 (27.0) | 0.068 | 355 (25.4) | 361 (25.8) | 0.015 |
| SVO | 395 (27.7) | 622 (29.1) |  | 388 (27.8) | 379 (27.1) |  |
| Other types e | 673 (47.2) | 937 (43.9) |  | 654 (46.8) | 657 (47.0) |  |
| **Infarction size, n(%)** |  |  |  |  |  |  |
| LI | 1079 (75.7) | 1562 (73.2) | 0.059 | 1056 (75.6) | 1058 (75.7) | 0.003 |
| FLI | 346 (24.3) | 573 (26.8) |  | 341 (24.4) | 339 (24.3) |  |
| **Baseline NIHSS score, n(%)** |  |  |  |  |  |  |
| ≤ 4 | 1008 (70.7) | 1528 (71.6) | 0.019 | 993 (71.1) | 999 (71.5) | 0.018 |
| 5-20 | 402 (28.2) | 586 (27.4) |  | 389 (27.8) | 381 (27.3) |  |
| ≥ 21 | 15 (1.1) | 21 (1.0) |  | 15 (1.1) | 17 (1.2) |  |
| **Laboratory parameters, (%)** f |  |  |  |  |  |  |
| WBC | 1392 (97.7) | 2087 (97.8) | 0.005 | 1366 (97.8) | 1367 (97.9) | 0.005 |
| NC | 1393 (97.8) | 2087 (97.8) | 0.000 | 1367 (97.9) | 1368 (97.9) | 0.005 |
| HGB | 1395 (97.9) | 2096 (98.2) | 0.020 | 1372 (98.2) | 1374 (98.4) | 0.011 |
| PLT | 1394 (97.8) | 2089 (97.8) | 0.001 | 1370 (98.1) | 1371 (98.1) | 0.005 |
| HCY | 1048 (73.5) | 1657 (77.6) | 0.095 | 1035 (74.1) | 1046 (74.9) | 0.018 |
| D-D | 1184 (83.1) | 1778 (83.3) | 0.005 | 1158 (82.9) | 1168 (83.6) | 0.019 |
| ALB | 1382 (97.0) | 2083 (97.6) | 0.036 | 1357 (97.1) | 1357 (97.1) | 0.000 |
| Cr | 1310 (91.9) | 2012 (94.2) | 0.091 | 1302 (93.2) | 1298 (92.9) | 0.011 |
| UA | 1349 (94.7) | 2005 (93.9) | 0.033 | 1323 (94.7) | 1323 (94.7) | 0.000 |
| TG | 1197 (84.0) | 1685 (78.9) | 0.131 | 1172 (83.9) | 1171 (83.8) | 0.002 |
| TC | 1311 (92.0) | 1917 (89.8) | 0.077 | 1286 (92.1) | 1273 (91.1) | 0.034 |
| HbA1c | 1050 (73.7) | 1569 (73.5) | 0.004 | 1027 (73.5) | 1025 (73.4) | 0.003 |

a Values are presented as n (%) or mean (SD).
b The SMD was used to compare characteristics between the DHI and Non-DHI groups, with an SMD < 0.1 indicating balanced and comparable covariates.
c “District” refers to Eastern (Zhejiang, Nanjing), Central (Shaanxi, Henan, Hunan), or Western (Guizhou, Yunnan) regions.
d “Stroke” includes ischemic stroke and hemorrhagic stroke; “Heart disease” includes coronary artery disease, myocardial infarction, atrial fibrillation, and heart failure.
e “Other types” refers to all AIS patients other than LAA and SVO.

f Laboratory tests were defined as a binary variable (1 = normal or abnormal with no clinical significance, 0 = abnormal with clinical significance). The table displays the number and proportion of patients classified as 1.

Abbreviations: DHI, Danhong Injection; SMD, standardized mean difference; SD, standard deviation; LAA, large-artery atherosclerosis; SVO, small-vessel occlusion; LI, lacunar infarction; FLI, focal or large-area infarction; NIHSS, National Institutes of Health Stroke Scale; WBC, white blood cell count; NC, neutrophil count; HGB, hemoglobin; PLT, platelet count; HCY, homocysteine; D-D, D-dimer; ALB, albumin; Cr, creatinine; CCr, creatinine clearance rate; UA, uric acid; TG, triglycerides; TC, total cholesterol; HbA1c, glycated hemoglobin.

**Table S4** Detailed definitions and amount of missing data.

|  | Normal or abnormal with no clinical significance | Abnormal with clinical significance | Missing data (%) |
| --- | --- | --- | --- |
| WBC | (3.5-12.0) × 10^9^/L | < 3.5×10^9^/L or > 12.0×10^9^/L | 0.08 |
| NC | (1.5-8.0) × 10^9^/L | < 1.5×10^9^/L or > 8.0×10^9^/L | 0.08 |
| HGB | ≥ 90g/L | < 90g/L | 0.08 |
| PLT | ≥ 100×10^9^/L | < 100×10^9^/L | 0.08 |
| HCY | ≤ 20μmol/L | > 20μmol/L | 17.87 |
| D-D | ≤ 1mg/L | > 1mg/L | 15.98 |
| ALB | ≥ 30g/L | < 30g/L | 0.20 |
| Cr | Cr < 133μmol/L and CCr > 80ml/min | Cr > 133μmol/L or CCr < 80ml/min | 0.11 |
| UA | ≤ 500μmol/L | > 500μmol/L | 0.22 |
| TG | ≤ 2.3mmol/L | > 2.3mmol/L | 2.42 |
| TC | ≤ 6.19mmol/L | > 6.19mmol/L | 2.42 |
| HbA1c | ≤ 6.5% | > 6.5% | 15.11 |

Except for the laboratory tests, all variables in this study were complete. Abbreviations: WBC, white blood cell count; NC, neutrophil count; HGB, hemoglobin; PLT, platelet count; HCY, homocysteine; D-D, D-dimer; ALB, albumin; Cr, creatinine; CCr, creatinine clearance rate; UA, uric acid; TG, triglycerides; TC, total cholesterol; HbA1c, glycated hemoglobin.

**Table S5** Sensitivity analysis of the associations between DHI use and the primary outcome.

| Analysis | DHI group a | Non-DHI group a | RR (95% CI) | *p*-value | MD b |
| --- | --- | --- | --- | --- | --- |
| Model 1 c | 2.02 (3.10) | 2.52 (3.31) | 0.80 (0.74-0.87) | < 0.001 | 0.59 (2.95) |
| Model 3 d | 1.99 (2.93) | 2.49 (3.30) | 0.80 (0.74-0.87) | < 0.001 | 0.68 (2.62) |
| Model 4 e | 1.96 (2.89) | 2.50 (3.37) | 0.78 (0.72-0.86) | < 0.001 | 0.62 (2.36) |
| Model 5 f | 1.82 (2.32) | 2.29 (2.56) | 0.80 (0.73-0.86) | < 0.001 | 0.56 (2.42) |

a Shown is the post-treatment NIHSS score, presented as mean (SD).
b MD is calculated as the difference in NIHSS (pre- to post-treatment) for the DHI group minus the difference in NIHSS (pre- to post-treatment) for the Non-DHI group, and is also presented as mean (SD).

c Model 1 includes sex, age, district, lifestyle, and previous medical history.
d Model 3 includes sex, age, district, lifestyle, previous medical history, disease course, TOAST classification, infarction size, baseline NIHSS score, and laboratory parameters.
e Model 4 excludes patients who received intravenous thrombolysis or endovascular therapy.
f Model 5 excludes patients with severe stroke (baseline NIHSS score ≥ 21).

**Table S6** Baseline characteristics of patients receiving or not receiving DHI after sIPTW.

| **Characteristics** | DHI group  (n=1424.8) | Non-DHI group  (n=2135.1) | SMD |
| --- | --- | --- | --- |
| **Sex, n(%)** |  |  |  |
| Female | 888.8 (62.4) | 1330.9 (62.3) | 0.001 |
| **Age, mean (SD)** | 67.82 (11.83) | 67.87 (11.54) | 0.004 |
| **District, n(%)** |  |  |  |
| East | 427.0 (30.0) | 642.0 (30.1) | 0.002 |
| Middle | 456.3 (32.0) | 682.2 (32.0) |  |
| West | 541.5 (38.0) | 811.0 (38.0) |  |
| **Smoking status, n(%)** |  |  |  |
| Current smoker | 400.1 (28.1) | 603.0 (28.2) | 0.004 |
| Former smoker | 118.9 (8.3) | 176.2 (8.3) |  |
| Never smoker | 905.8 (63.6) | 1355.9 (63.5) |  |
| **Drinking, n (%)** | 358.6 (25.2) | 541.9 (25.4) | 0.005 |
| **Medical history, n(%)** |  |  |  |
| Stroke | 478.1 (33.6) | 717.6 (33.6) | 0.001 |
| Ischemic stroke | 449.9 (31.6) | 674.5 (31.6) | < 0.001 |
| Heart disease | 312.0 (21.9) | 468.6 (21.9) | 0.001 |
| Hypertension | 1121.6 (78.7) | 1681.6 (78.8) | 0.001 |
| Type 2 diabetes | 510.1 (35.8) | 763.9 (35.8) | < 0.001 |
| Hyperlipidemia | 377.3 (26.5) | 566.5 (26.5) | 0.001 |
| **Disease course, n(%)** |  |  |  |
| ≤ 1d | 732.1 (51.4) | 1098.9 (51.5) | 0.002 |
| **TOAST classification, n(%)** |  |  |  |
| LAA | 373.2 (26.2) | 559.4 (26.2) | 0.002 |
| SVO | 409.3 (28.7) | 611.4 (28.6) |  |
| Other types | 642.3 (45.1) | 964.4 (45.2) |  |
| **Infarction size, n(%)** |  |  |  |
| LI | 1060.0 (74.4) | 1585.5 (74.3) | 0.003 |
| **Baseline NIHSS score, n(%)** |  |  |  |
| ≤ 4 | 1015.9 (71.3) | 1521.0 (71.2) | 0.002 |
| 5-20 | 394.3 (27.7) | 592.4 (27.7) |  |
| ≥ 21 | 14.6 (1.0) | 21.8 (1.0) |  |

The weighted number of cases was calculated based on sIPTW, reflecting a statistically adjusted sample size rather than the actual count of patients. Therefore, non-integer values may arise, which is a normal characteristic of this weighting approach. Abbreviations: sIPTW, stable inverse probability of treatment weighting.

**Table S7** Baseline characteristics of patients receiving or not receiving DHI before and after propensity-score matching (Model 1).

| **Characteristics** a | Unmatched patients | | | Propensity-score-matched patients | | |
| --- | --- | --- | --- | --- | --- | --- |
|  | DHI Group  (n = 1425) | Non-DHI Group  (n = 2135) | SMD b | DHI Group  (n = 1420) | Non-DHI Group  (n = 1420) | SMD |
| **Sex, n(%)** |  | | | | | |
| Male | 883 (62.0) | 1334 (62.5) | 0.011 | 880 (62.0) | 887 (62.5) | 0.010 |
| Female | 542 (38.0) | 801 (37.5) |  | 540 (38.0) | 533 (37.5) |  |
| **Age, mean (SD)** | 68.37 (11.84) | 67.61 (11.55) | 0.065 | 68.35 (11.84) | 68.11 (11.44) | 0.021 |
| **District, n(%)** c |  | | | | | |
| East | 479 (33.6) | 594 (27.8) | 0.178 | 474 (33.4) | 463 (32.6) | 0.018 |
| Middle | 476 (33.4) | 660 (30.9) |  | 476 (33.5) | 477 (33.6) |  |
| West | 470 (33.0) | 881 (41.3) |  | 470 (33.1) | 480 (33.8) |  |
| **Smoking status, n(%)** |  | | | | | |
| Current smoker | 392 (27.5) | 615 (28.8) | 0.046 | 389 (27.4) | 389 (27.4) | 0.005 |
| Former smoker | 110 (7.7) | 182 (8.5) |  | 110 (7.7) | 112 (7.9) |  |
| Never smoker | 923 (64.8) | 1338 (62.7) |  | 921 (64.9) | 919 (64.7) |  |
| **Drinking, n (%)** | 345 (24.2) | 562 (26.3) | 0.049 | 341 (24.0) | 347 (24.4) | 0.010 |
| **Medical history, n(%)** d |  | | | | | |
| Stroke | 525 (36.8) | 671 (31.4) | 0.114 | 520 (36.6) | 524 (36.9) | 0.006 |
| Ischemic stroke | 504 (35.4) | 620 (29.0) | 0.136 | 499 (35.1) | 503 (35.4) | 0.006 |
| Heart disease | 332 (23.3) | 451 (21.1) | 0.052 | 330 (23.2) | 339 (23.9) | 0.015 |
| Hypertension | 1103 (77.4) | 1704 (79.8) | 0.059 | 1098 (77.3) | 1102 (77.6) | 0.007 |
| Type 2 diabetes | 508 (35.6) | 766 (35.9) | 0.005 | 507 (35.7) | 523 (36.8) | 0.023 |
| Hyperlipidemia | 341 (23.9) | 605 (28.3) | 0.100 | 341 (24.0) | 321 (22.6) | 0.033 |

a Values are presented as n (%) or mean (SD).
b The SMD was used to compare characteristics between the DHI and Non-DHI groups, with an SMD < 0.1 indicating balanced and comparable covariates.
c “District” refers to Eastern (Zhejiang, Nanjing), Central (Shaanxi, Henan, Hunan), or Western (Guizhou, Yunnan) regions.
d “Stroke” includes ischemic stroke and hemorrhagic stroke; “Heart disease” includes coronary artery disease, myocardial infarction, atrial fibrillation, and heart failure.

**Table S8** Baseline characteristics before and after PSM, excluding patients who received intravenous thrombolysis or endovascular therapy (Model 4).

| **Characteristics** a | Unmatched patients | | | Propensity-score-matched patients | | |
| --- | --- | --- | --- | --- | --- | --- |
|  | DHI group  (n = 1362) | Non-DHI group  (n = 2034) | SMD b | DHI group  (n = 1354) | Non-DHI group  (n = 1354) | SMD |
| **Sex, n(%)** |  | | | | | |
| Male | 839 (61.6) | 1275 (62.7) | 0.022 | 834 (61.6) | 846 (62.5) | 0.018 |
| Female | 523 (38.4) | 759 (37.3) |  | 520 (38.4) | 508 (37.5) |  |
| **Age, mean (SD)** | 68.43 (11.83) | 67.69 (11.58) | 0.063 | 68.42 (11.84) | 68.14 (11.55) | 0.024 |
| **District, n(%)** c |  | | | | | |
| East | 456 (33.5) | 558 (27.4) | 0.182 | 448 (33.1) | 447 (33.0) | 0.037 |
| Middle | 453 (33.3) | 627 (30.8) |  | 453 (33.5) | 433 (32.0) |  |
| West | 453 (33.3) | 849 (41.7) |  | 453 (33.5) | 474 (35.0) |  |
| **Smoking status, n(%)** |  | | | | | |
| Current smoker | 372 (27.3) | 584 (28.7) | 0.047 | 370 (27.3) | 383 (28.3) | 0.024 |
| Former smoker | 107 (7.9) | 176 (8.7) |  | 107 (7.9) | 102 (7.5) |  |
| Never smoker | 883 (64.8) | 1274 (62.6) |  | 877 (64.8) | 869 (64.2) |  |
| **Drinking, n (%)** | 330 (24.2) | 540 (26.5) | 0.053 | 329 (24.3) | 323 (23.9) | 0.010 |
| **Medical history, n(%)** d |  | | | | | |
| Stroke | 503 (36.9) | 648 (31.9) | 0.107 | 497 (36.7) | 488 (36.0) | 0.014 |
| Ischemic stroke | 482 (35.4) | 598 (29.4) | 0.128 | 476 (35.2) | 474 (35.0) | 0.003 |
| Heart disease | 307 (22.5) | 421 (20.7) | 0.045 | 304 (22.5) | 306 (22.6) | 0.004 |
| Hypertension | 1062 (78.0) | 1625 (79.9) | 0.047 | 1057 (78.1) | 1057 (78.1) | 0.000 |
| Type 2 diabetes | 492 (36.1) | 731 (35.9) | 0.004 | 486 (35.9) | 515 (38.0) | 0.044 |
| Hyperlipidemia | 326 (23.9) | 574 (28.2) | 0.098 | 326 (24.1) | 332 (24.5) | 0.010 |
| **Disease course, n(%)** |  | | | | | |
| ≤ 1d | 653 (47.9) | 1025 (50.4) | 0.049 | 648 (47.9) | 638 (47.1) | 0.015 |
| > 1d | 709 (52.1) | 1009 (49.6) |  | 706 (52.1) | 716 (52.9) |  |
| **TOAST classification, n(%)** |  | | | | | |
| LAA | 328 (24.1) | 528 (26.0) | 0.067 | 328 (24.2) | 327 (24.2) | 0.015 |
| SVO | 387 (28.4) | 607 (29.8) |  | 386 (28.5) | 395 (29.2) |  |
| Other types e | 647 (47.5) | 899 (44.2) |  | 640 (47.3) | 632 (46.7) |  |
| **Infarction size, n(%)** |  |  |  |  |  |  |
| LI | 1059 (77.8) | 1527 (75.1) | 0.063 | 1053 (77.8) | 1054 (77.8) | 0.002 |
| FLI | 303 (22.2) | 507 (24.9) |  | 301 (22.2) | 300 (22.2) |  |
| **Baseline NIHSS score, n(%)** |  |  |  |  |  |  |
| ≤ 4 | 986 (72.4) | 1488 (73.2) | 0.024 | 983 (72.6) | 974 (71.9) | 0.019 |
| 5-20 | 362 (26.6) | 529 (26.0) |  | 357 (26.4) | 364 (26.9) |  |
| ≥ 21 | 14 (1.0) | 17 (0.8) |  | 14 (1.0) | 16 (1.2) |  |

a Values are presented as n (%) or mean (SD).
b The SMD was used to compare characteristics between the DHI and Non-DHI groups, with an SMD < 0.1 indicating balanced and comparable covariates.
c “District” refers to Eastern (Zhejiang, Nanjing), Central (Shaanxi, Henan, Hunan), or Western (Guizhou, Yunnan) regions.
d “Stroke” includes ischemic stroke and hemorrhagic stroke; “Heart disease” includes coronary artery disease, myocardial infarction, atrial fibrillation, and heart failure.
e “Other types” refers to all AIS patients other than LAA and SVO.

**Table S9** Baseline characteristics before and after PSM, excluding patients with severe stroke (baseline NIHSS score ≥ 21) (Model 5).

| **Characteristics** a | Unmatched patients | | | Propensity-score-matched patients | | |
| --- | --- | --- | --- | --- | --- | --- |
|  | DHI group  (n = 1410) | Non-DHI group  (n = 2114) | SMD b | DHI group  (n = 1396) | Non-DHI group  (n = 1396) | SMD |
| **Sex, n(%)** |  | | | | | |
| Male | 876 (62.1) | 1325 (62.7) | 0.011 | 865 (62.0) | 873 (62.5) | 0.012 |
| Female | 534 (37.9) | 789 (37.3) |  | 531 (38.0) | 523 (37.5) |  |
| **Age, mean (SD)** | 68.32 (11.83) | 67.57 (11.54) | 0.064 | 68.23 (11.81) | 68.09 (11.48) | 0.011 |
| **District, n(%)** c |  | | | | | |
| East | 468 (33.2) | 589 (27.9) | 0.174 | 454 (32.5) | 461 (33.0) | 0.020 |
| Middle | 474 (33.6) | 649 (30.7) |  | 474 (34.0) | 461 (33.0) |  |
| West | 468 (33.2) | 876 (41.4) |  | 468 (33.5) | 474 (34.0) |  |
| **Smoking status, n(%)** |  | | | | | |
| Current smoker | 389 (27.6) | 610 (28.9) | 0.046 | 384 (27.5) | 379 (27.1) | 0.028 |
| Former smoker | 109 (7.7) | 181 (8.6) |  | 108 (7.7) | 99 (7.1) |  |
| Never smoker | 912 (64.7) | 1323 (62.6) |  | 904 (64.8) | 918 (65.8) |  |
| **Drinking, n (%)** | 343 (24.3) | 557 (26.3) | 0.046 | 340 (24.4) | 339 (24.3) | 0.002 |
| **Medical history, n(%)** d |  | | | | | |
| Stroke | 520 (36.9) | 659 (31.2) | 0.121 | 510 (36.5) | 511 (36.6) | 0.001 |
| Ischemic stroke | 500 (35.5) | 608 (28.8) | 0.144 | 490 (35.1) | 489 (35.0) | 0.002 |
| Heart disease | 326 (23.1) | 444 (21.0) | 0.051 | 321 (23.0) | 336 (24.1) | 0.025 |
| Hypertension | 1092 (77.4) | 1689 (79.9) | 0.060 | 1083 (77.6) | 1099 (78.7) | 0.028 |
| Type 2 diabetes | 505 (35.8) | 758 (35.9) | 0.001 | 498 (35.7) | 501 (35.9) | 0.004 |
| Hyperlipidemia | 340 (24.1) | 601 (28.4) | 0.098 | 340 (24.4) | 335 (24.0) | 0.008 |
| **Disease course, n(%)** |  | | | | | |
| ≤ 1d | 703 (49.9) | 1106 (52.3) | 0.049 | 697 (49.9) | 678 (48.6) | 0.027 |
| > 1d | 707 (50.1) | 1008 (47.7) |  | 699 (50.1) | 718 (51.4) |  |
| **TOAST classification, n(%)** |  | | | | | |
| LAA | 346 (24.5) | 557 (26.3) | 0.064 | 345 (24.7) | 352 (25.2) | 0.012 |
| SVO | 395 (28.0) | 621 (29.4) |  | 392 (28.1) | 392 (28.1) |  |
| Other types e | 669 (47.4) | 936 (44.3) |  | 659 (47.2) | 652 (46.7) |  |
| **Infarction size, n(%)** |  |  |  |  |  |  |
| LI | 1079 (76.5) | 1560 (73.8) | 0.063 | 1067 (76.4) | 1066 (76.4) | 0.002 |
| FLI | 331 (23.5) | 554 (26.2) |  | 329 (23.6) | 330 (23.6) |  |
| **Baseline NIHSS score, n(%)** |  |  |  |  |  |  |
| ≤ 4 | 1008 (71.5) | 1528 (72.3) | 0.018 | 1001 (71.7) | 996 (71.3) | 0.008 |
| 5-20 | 402 (28.5) | 586 (27.7) |  | 395 (28.3) | 400 (28.7) |  |

a Values are presented as n (%) or mean (SD).
b The SMD was used to compare characteristics between the DHI and Non-DHI groups, with an SMD < 0.1 indicating balanced and comparable covariates.
c “District” refers to Eastern (Zhejiang, Nanjing), Central (Shaanxi, Henan, Hunan), or Western (Guizhou, Yunnan) regions.
d “Stroke” includes ischemic stroke and hemorrhagic stroke; “Heart disease” includes coronary artery disease, myocardial infarction, atrial fibrillation, and heart failure.
e “Other types” refers to all AIS patients other than LAA and SVO.


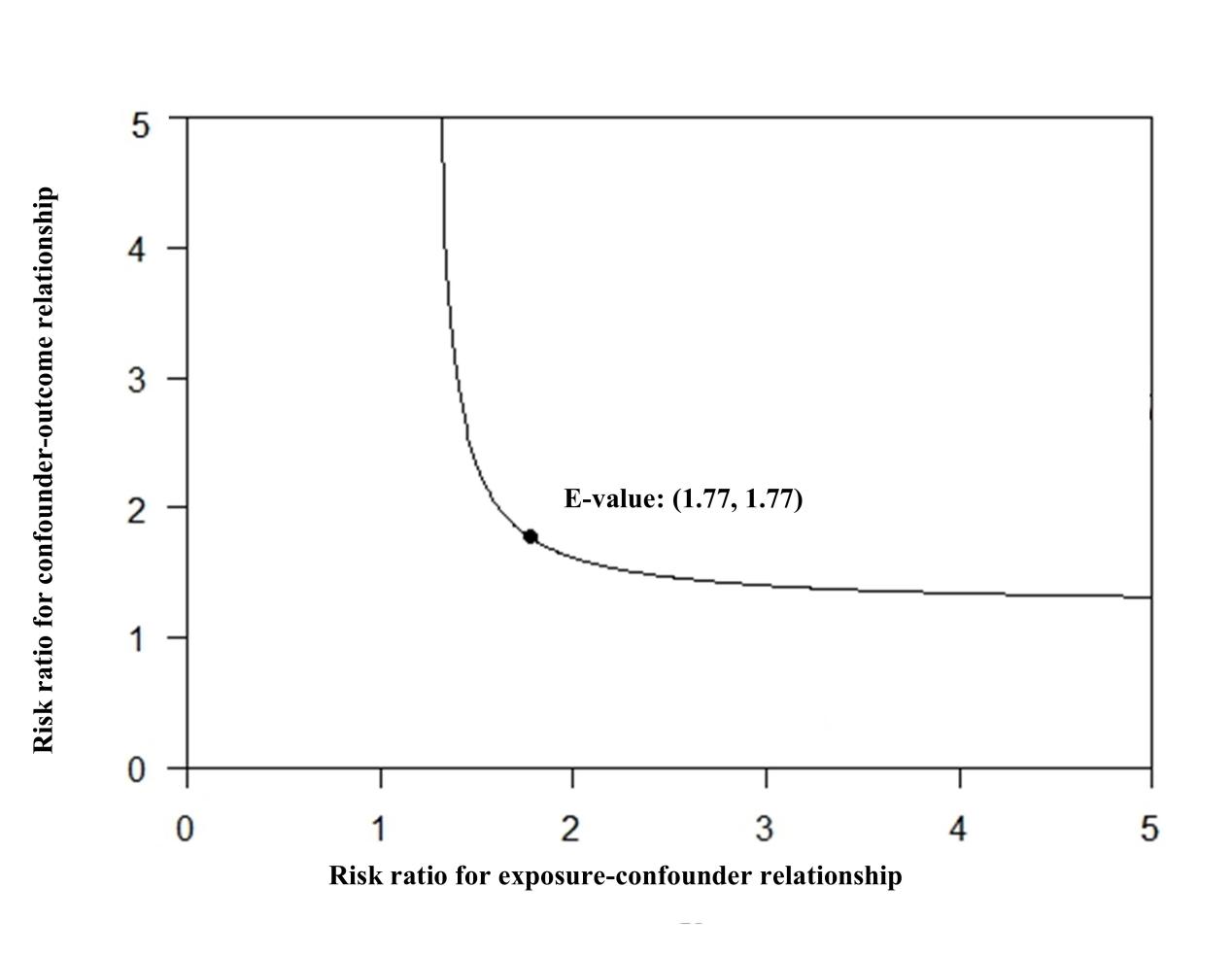


**Figure S1** Post-hoc E-value analysis to assess the extent of unmeasured confounding that would be required to negate the observed results.


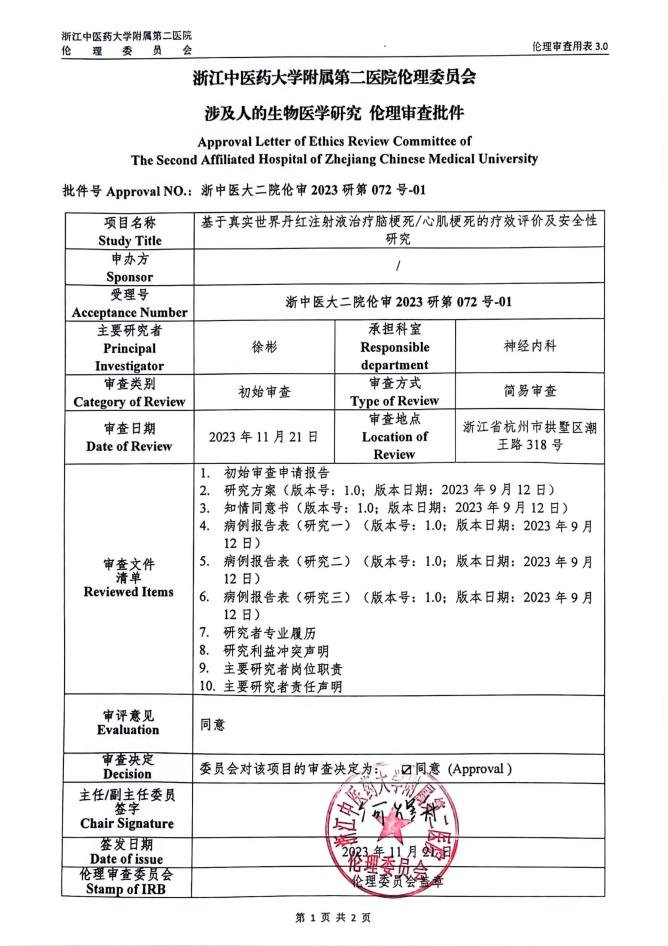

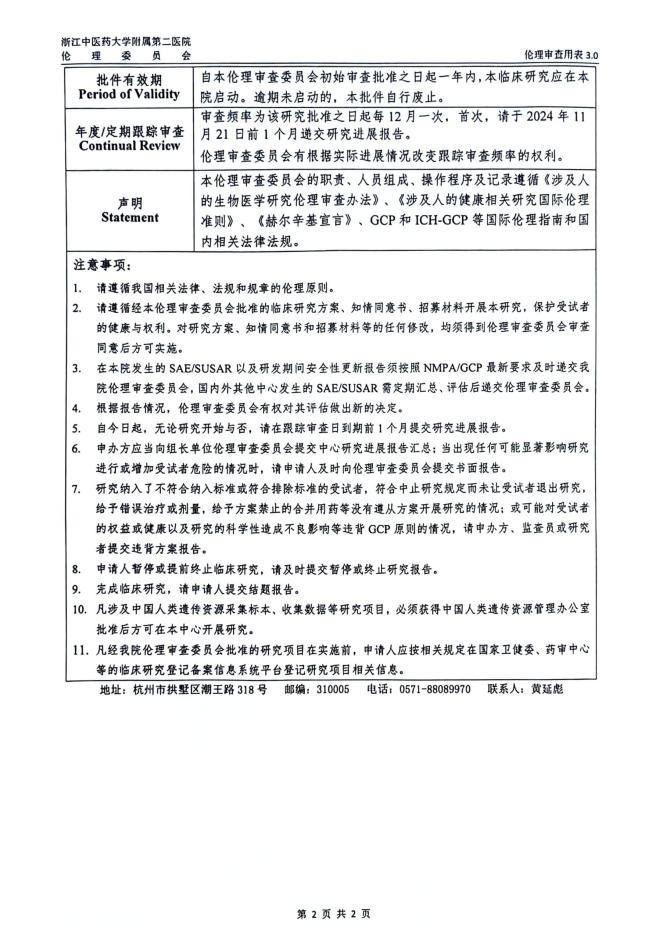


**Figure S2** Ethics approval document.
